# Training Computers to See the Built Environment Related to Physical Activity: Detection of Microscale Walkability Features Using Computer Vision

**DOI:** 10.3390/ijerph19084548

**Published:** 2022-04-09

**Authors:** Marc A. Adams, Christine B. Phillips, Akshar Patel, Ariane Middel

**Affiliations:** 1 College of Health Solutions, Arizona State University, Phoenix, AZ 85004, USA; aksharpatel@asu.edu; 2 Department of Psychology, Clemson University, Clemson, SC 29634, USA; cbphill@clemson.edu; 3 Herberger Institute for Design and the Arts, School of Arts, Media and Engineering, Arizona State University, Phoenix, AZ 85004, USA; ariane.middel@asu.edu

**Keywords:** built environment, computer vision, deep learning, Google Street View, microscale, walkability

## Abstract

The study purpose was to train and validate a deep learning approach to detect microscale streetscape features related to pedestrian physical activity. This work innovates by combining computer vision techniques with Google Street View (GSV) images to overcome impediments to conducting audits (e.g., time, safety, and expert labor cost). The EfficientNETB5 architecture was used to build deep learning models for eight microscale features guided by the Microscale Audit of Pedestrian Streetscapes Mini tool: sidewalks, sidewalk buffers, curb cuts, zebra and line crosswalks, walk signals, bike symbols, and streetlights. We used a train–correct loop, whereby images were trained on a training dataset, evaluated using a separate validation dataset, and trained further until acceptable performance metrics were achieved. Further, we used trained models to audit participant (*N* = 512) neighborhoods in the WalkIT Arizona trial. Correlations were explored between microscale features and GIS-measured and participant-reported neighborhood macroscale walkability. Classifier precision, recall, and overall accuracy were all over >84%. Total microscale was associated with overall macroscale walkability (*r* = 0.30, *p* < 0.001). Positive associations were found between model-detected and self-reported sidewalks (*r* = 0.41, *p* < 0.001) and sidewalk buffers (*r* = 0.26, *p* < 0.001). The computer vision model results suggest an alternative to trained human raters, allowing for audits of hundreds or thousands of neighborhoods for population surveillance or hypothesis testing.

## 1. Introduction

The health and wellbeing benefits of physical activity and its environmental and economic co-benefits are well established [1]. Ecological models posit and evidence consistently shows that approaches for promoting physical activity must address multiple levels of influence, including built environments. Features of the built environment can influence physical activity behaviors directly through accessibility, pedestrian safety, comfort, and the affective experiences of walking and active travel [1,2,3,4,5,6,7]. In the context of behavioral interventions, evidence suggests the built environment interacts with intervention components to impact physical activity adoption and maintenance [3,8,9,10,11]. A supportive built environment can facilitate walking and active travel [6,11], while an unsupportive environment may be a barrier to physical activity engagement [6,11], warranting behavioral intervention to overcome [8,9].

Features supportive of walking and active travel can be measured at the macroscale using geographic information systems (GIS) or at the microscale (street-level) with in-person streetscape audits, and either may be closer to measuring what exists than perceptions [12]. Features measured on a macroscale, such as land-use mix, intersection density, park, transit, or residential density, are generally difficult to modify but are easily assessed using publicly available data sources. Microscale built environment features such as pedestrian amenities that increase the safety and comfort of active travel can explain further variance in physical activity, even after adjusting for macroscale walkability [4,13].

Microscale features are more cost-effectively modified than macroscale features but are measured much less often. This observation is likely due to the limited feasibility of conducting neighborhood streetscape audits. Traditional in-person microscale audits are expensive, require extensive travel and audit time, and expose auditors to crime, traffic, and weather safety concerns. As a result, relationships among microscale neighborhood environments, physical activity, and the shared health, environmental, and economic co-benefits of active living [1] are not broadly researched. Substantial population differences may be linked to relatively inexpensive and easily modifiable street-level features, but evidence to show inequities across neighborhoods is lacking.

Virtual microscale audits by human raters using online mapping tools such as Google Street View (GSV) to scroll down and audit a streetscape are reliable alternatives to traditional in-person audits [14,15,16,17,18,19,20,21,22]. Virtual audits effectively eliminate travel, weather, and safety challenges of standard in-person audits. However, virtual audits conducted by human content experts remain time-intensive [23,24,25], are limited to small areas or short routes [4,21], require extensive auditor training and retraining, and are susceptible to auditor fatigue [26]. Thus, the scalability of virtual audits remains dependent on the amount of available trained human labor. The challenge of scaling in-person or virtual audits to assess hundreds or thousands of neighborhoods continues to be a primary obstacle to surveillance (e.g., changes over time) or hypothesis testing.

Combining computer-enabled deep learning and computer vision techniques is an emerging approach for increasing the scalability of collecting street-level environmental data. Deep learning is a subset of artificial intelligence that uses algorithms (i.e., neural networks) to learn to recognize and interpret patterns in data. The algorithms used in deep learning are self-adaptive, meaning that the networks get smarter when given more training data or training time. Computer vision is a broad term describing how computers see and understand digital visual data. When deep learning is combined with computer vision, neural network models can be trained to recognize built environment patterns in GSV images for classification tasks [27] such as land-use (e.g., building classification [28], scene classification (e.g., perceived streetscape safety [29]) and object detection (e.g., detecting and classifying automobiles [30] or cataloging trees [31]).

Among classification tasks, image classification using neural networks has been the most commonly used approach for remotely detecting specific features present in GSV images. For example, Hara and colleagues combined crowdsourcing with deep learning and computer vision to detect curb ramps in GSV imagery to assess sidewalk accessibility for disabled individuals [32]. Since then, others have worked to automate the detection of curb cuts [33], crosswalks [34], and other built environment objects visible in GSV images [35,36,37]. For example, Koo et al. selected eight of 150 categories available from the existing Pyramid Scene Parsing Network (PSPN) model to represent mesoscale streetscapes including building, house, sidewalk, tree, road, grass, car, and plant [38]. They calculated three indices from these categories: building-to-street ratio, greenness, and sidewalk-to-street proportion, and they found that the building-to-street ratio and greenness were associated with reported walking trips. These advances have led to the promise of developing an automated or semi-automated approach for conducting pedestrian streetscape audits. However, the development of automated microscale tools remains an open problem. Existing research relies heavily on deep learning models developed for broad classifications, which were not developed or validated for features related to physical activity. Furthermore, custom trained models for physical activity-related behaviors focused on a small number of microscale features (e.g., curb ramps), trained and/or validated models in one geographic region (e.g., Atlanta), did not examine how model-detected features align with perceptions of features (or feature indices) by individuals living in evaluated neighborhoods, or suffered some combinations of these issues. Developing a reliable and validated automated tool for detecting microscale features that reduces reliance on human labor is crucial to addressing current issues of scalability. Increasing scalability will enable greater numbers of studies investigating the influence of microscale built environment features, as well as including larger and more diverse samples of participants and neighborhoods to allow generalizability.

The current study explores whether eight microscale features selected mainly from an existing validated tool, the Microscale Audit of Pedestrian Streetscapes (MAPS) Mini [13], could be reliably trained and validated using computer vision and deep learning techniques from a sample of GSV images across five cities. We further inferred the presence or absence of these microscale features in GSV images within buffers around 512 homes for participants enrolled in the baseline phase of the WalkIT Arizona physical activity trial. Additionally, we examined the correlations between our automated micro-scale audit against macroscale walkability and participant perceptions of these neighborhood features and related subscales.

This paper is structured as follows: in Section 2 (Materials and Methods), we introduce the study design and participant recruitment for the walkability analysis, outline how perceived walkability is measured, and detail how macroscale walkability is determined using GIS. For the microscale walkability assessment using GSV images, we present how microscale features are derived from the images using training and validation datasets and outline the classifier training and evaluation process. We then explain how neighborhood microscale features are quantified and analyzed. In Section 3 (Results), we present the image classifier performance and the model inference results. Lastly, we discuss the results in Section 4 and offer conclusions.

## 2. Materials and Methods

*Study Design and Participant Recruitment*. The current study used data collected from participants enrolled in the WalkIT Arizona trial as described by Adams et al. [10]. Briefly, participant enrollment was balanced across four neighborhood types in Maricopa County, AZ, according to census block group socioeconomic status (SES) and GIS-measured macro-level walkability. For participant sampling, we computed block group SES and walkability using available census median income data and public regional datasets for net residential density, land use, intersection density, and public transit density. Following Frank et al. [6], block groups in the first through fifth deciles of SES were categorized as “lower SES” and those in the seventh through 10th deciles were categorized as “higher SES”. The sixth decile was omitted to minimize mis-categorization for participants on the boundaries. Similarly, block groups were ranked and categorized into “lower walkable” (first through fourth deciles) and “higher walkable” (seventh through 10th deciles) with the fifth and sixth walkability deciles excluded to minimize the likelihood of mis-categorization. Finally, block groups were classified according to their combined walkability and SES yielding four neighborhood strata: “higher SES/higher walkable”, “lower SES/higher walkable”, “higher SES/lower walkable”, and “lower SES/lower walkable”. Study marketing materials targeted eligible block groups from these strata.

Enrolled participants (*N* = 512) met the following inclusion criteria: (1) lived in one of the four neighborhood strata in Maricopa County, (2) 18–60 years of age, (3) generally healthy, and (4) insufficiently active. The number of participants for each neighborhood type ranged from 108 in the “lower walkable/lower SES” to 136 in the “higher walkable/higher income” and “lower walkable/higher income” neighborhoods. The mean age was 45.5 (SD = 9.1) years, with the majority of the sample reporting being female (64.3%), white (84%), non-Hispanic or Latino (81.2%), and married or living with a partner (67.5%). The sample reported a median household income of 60,000–79,999 USD, median educational attainment of college graduate, a median distance to work of 10.1 miles (16,316 m), and a median time at current residence of 52 months (see Adams et al. for full inclusion/exclusion criteria and sample characteristics [10]).

*Perceived Walkability Attributes*. Participants evaluated their perceived neighborhood attributes using the Neighborhood Environment Walkability Scale (NEWS) [39], completed at the baseline appointment. Seven NEWS subscales were computed using scoring guidelines published at https://drjimsallis.org/Documents/Measures_documents/NEWS_scoring.pdf (accessed on 14 March 2015). These included residential density, proximity to nonresidential land uses, street connectivity, presence of walking and cycling facilities, aesthetics, traffic safety, and crime safety. Higher scores on each of the subscales and index score indicate higher perceived walkability. The NEWS subscales have demonstrated good to excellent test–retest reliability and the ability to discriminate between high and low walkable neighborhoods [40,41].

**Figure 1:**
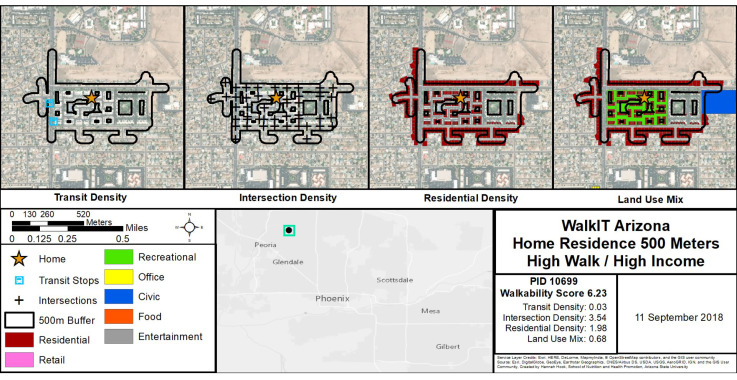
Example of individual-level buffers and macroscale walkability components and index values for a single participant’s neighborhood. Note: In the example in Figure 1, land-use mix shows residential, recreational, and civic uses. Other land uses such as office, food, entertainment, and retail were possible but not present in this example.

*GIS-Measured Macroscale Walkability*. In addition to block group walkability described above for recruitment and enrollment purposes, we also calculated individual-level walkability components and the overall index around enrolled participant homes. Participants’ home residential addresses were geocoded using ArcGIS 10.5 (ESRI, Redlands, CA, USA) with US Census Tigerline address feature. Geocoded addresses were used to create a 500 m “individual-level” buffer throughout the street network and to geoprocess spatial datasets and create “individual-level” GIS variables for the following components: net residential density (i.e., number of housing units divided by residential parcel land area), land-use mix (i.e., diversity of several land uses with normalized scores ranging from 0 for single use to 1 indicating an even distribution across residential, retail, recreational, office, civic, food, and entertainment parcel land uses), intersection density (i.e., number of three-leg or more intersections), and public transit access. A composite walkability index was calculated with the following formula: *walkability index* = [(*z*-score for net residential density) + (*z*-score for land-use mix) + (*z*-score for intersection density) + (*z*-score for transit access)]. Figure 1 provides a visual example of these variables.

*Microscale Features and Training and Validation Datasets*. To curate a high-quality dataset of labeled features for the microscale features of interest for training and validation, we relied on existing GSV images of street intersections from Phoenix, AZ (133,235 images), Washington, DC (20,784 images), San Diego, CA (8000 images), Seattle, WA (8000 images), and Baltimore, MD (8578 images). In addition, we relied on non-intersection images in Phoenix (1,331,994 images). The images were retrieved between 2018 and 2019 for urban climate studies [42,43,44]. Because images did not necessarily contain a feature of interest, we relied on a larger set of images than used for any single feature. The advantage of dividing the images into these intersection and non-intersection categories was to allow us to use only images that were necessary for a specific image classifier. For example, to train a zebra crosswalk classifier, we only needed intersection images, and, for sidewalks and sidewalk buffers, we required both intersection and non-intersection images. The following classes were used to create their respective image classifiers: (1) sidewalk (2) sidewalk buffer (3) curb cut (4) zebra crosswalk (5) line crosswalk (6) walk signal (7) bike symbol, and (8) streetlight.

**Figure 2:**
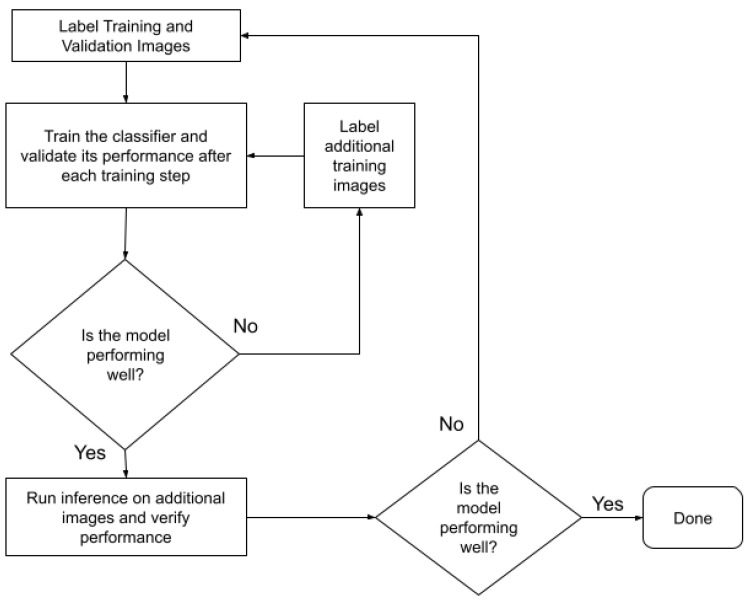
Classifier training and evaluation process.

*Creating Image Classifiers*. To study associations between model-detected microscale street features and GIS-measured and perceived neighborhood walkability, we wanted the system to determine the presence or absence of each of the eight street features at every audit point within participants’ neighborhood network buffers. To accomplish this, we created a separate image classifier for each street feature using the EfficientNetB5 neural architecture [45]. For each input image, the classifier output the probability of street feature presence (i.e., crosswalk, curb cut, etc.) using the visual features it identified in the image. The classifier training and evaluation process consisted of the steps shown in Figure 2 and further described below.

*Creating Initial Maricopa Datasets*. The first step in creating our classifiers was to create a set of images labeled with the appropriate classification (i.e., presence or absence of street feature) to train the classifier and a separate set of labeled images to validate classifier performance after each step of the training process. We used our knowledge of Phoenix neighborhoods to select initial GSV images from existing image datasets to be used in the training and validation datasets. To label the datasets, we used the open annotation tool, wkentaro/labelme, available at: https://zenodo.org/record/5711226#.YhzeS-jMJaQ (accessed on 10 April 2018) [46].

*Training and Validation Loop*: Using the initial training and validation datasets in place for each street feature, we trained a classifier to recognize the presence or absence of a street feature in an image. Each step in the classifier training process was one pass through the entire training dataset. After each step, the trained classifier was saved for potential future use. The training continued until the classifier started overfitting or the performance metrics did not improve after each step. To prevent overfitting, we tracked training and validation error values. We determined that the classifier had overfit when the training error continued to decrease but the validation error began to increase. When training was complete, we selected the classifier with the best performance metrics from all the saved classifiers while making sure that the classifier had not overfit [47].

For image sets with unsatisfactory performance metrics of the trained classifier, we visually examined the validation dataset to understand false positive and false negative results. We then found additional images from the Phoenix dataset that were similar to the ones where the classifier failed, labeled them, and added them to the training dataset. With the new training dataset, we restarted the training process.

Once the trained classifier performed well, we ran inference on additional Phoenix images to understand how the classifier was performing on those additional images. If the classifier identified the street features in the new images well, we considered that classifier to be trained. Otherwise, we tried to understand the images where the classifier failed, labeled the images, added those and other similar labeled images to the training and validation datasets, and restarted the training process.

We further improved the classifier by adding images from other cities (i.e., San Diego, Washington DC, Seattle, and Baltimore) to the training datasets.

*Considerations in Training the Classifiers*: *Single vs. Multiple Classifiers*. We selected an approach using a separate classifier for each feature instead of a single classifier that would simultaneously detect all features because it allowed us to iterate and improve on each feature classifier effectively and efficiently. Additionally, a single classifier approach can be problematic due to the discrepancy in prevalence across features. Training a single classifier to improve on a specific feature that is less prevalent in images, such as a zebra crosswalk, can lead to overfitting [47] on detecting a feature that is highly prevalent in the dataset such as a curb cut. Thus, a single-model approach may require settling on a poorer-performing model overall to balance these issues across features. 

*Selecting the Classifier Architecture*. Considering the results of how different neural network architectures performed on the ImageNet challenge [48], as well as the availability of pretrained weights, we decided to base our classifier on the EfficientNetB5 [45] architecture. 

*Selecting a Deep Learning Framework*. Frameworks such as TensorFlow, Keras, PyTorch, Caffe, and Fast.ai are popular in the deep learning field to create neural networks that solve a variety of computer vision problems. We evaluated the different frameworks for the purpose of creating image classifiers and selected Fast.ai [49] due to its ease of use, inbuilt data augmentation capabilities, and the simplicity of accomplishing transfer learning. 

*Transfer Learning*. Training a classifier as deep as EfficientNetB5 is a time-consuming process. To reduce classifier training time and quickly iterate to improve model performance, we used transfer learning. To achieve transfer learning, we used weights from a classifier pretrained on the ImageNet dataset as the initial weights for training our classifiers [50]. The pretrained classifiers could identify patterns for ~1000 different classes, which facilitated training our image classifiers. 

*Data Augmentation Techniques*. When selecting the data augmentation techniques, an important consideration was ensuring that augmenting did not result in losing information that was critical for the classifier to infer a feature. For example, if a sidewalk was only a small section of the image at one of the edges, zoom, crop, warp, cutout, and rotation augmentations could completely remove the sidewalk from the image, resulting in the classifier learning incomplete information. Thus, we applied only three data augmentation techniques: (1) horizontal flip, (2) brightness, and (3) contrast adjustments.

*Quantifying Neighborhood Microscale Features*. The trained models were used to infer (i.e., detect) eight street features in 765,869 previously unexamined photos available in participant buffers in Phoenix, AZ. The probability threshold to classify “presence” of a detected feature in images was set to ≥0.50 and used for each cardinal direction associated with every coordinate (i.e., if the model probability of specific feature presence in an image was ≥0.50, we classified the feature as present). To capture the presence of sidewalks on a block regardless of the side of the street, we averaged the four model-detected probabilities from the four directional images. This approach was used for sidewalks to ensure we did not miss sidewalks only on one side of the block or visible in only one of the four images. For sidewalk buffers, we estimated the presence of sidewalk buffers for coordinates with a model-detected sidewalk only. To summarize each participant’s neighborhood, we summed the count of coordinates with positive instances of each feature within the neighborhood buffer and divided by the count of coordinates within the buffer to obtain an average count of neighborhood coordinates with positive instances of each feature. Because the denominator (number of coordinates) could vary by intersection vs. non-intersection feature, the averages of each feature were *z*-scored to create a ranking relative to the sample mean. A “total microscale feature score” for each participant’s home neighborhood was created by summing individual *z*-scores for each of the microscale features detected within the neighborhood buffer.

*Analytic Plan*. The performance of the image classifier was assessed using the validation dataset for Phoenix, AZ. As the task was classifying images by street feature presence or absence, we calculated precision, recall, negative predictive value, specificity, and accuracy for each feature. Precision was the probability that, following a positive model-detected observation, the image truly had the feature present (i.e., true positives/true positives + false positives). Recall was the probability that a model-detected observation was truly present in an image (i.e., true positives/true positives + false negatives). Negative predictive value was the probability that, following a negative model-detected observation, the image truly did not have a feature present (i.e., true negatives/true negatives + false negatives). Specificity was the proportion of images classified as not having a feature among all images that truly did not have the feature present (i.e., true negatives/true negatives + false positives). Accuracy was the probability of a correct observation (i.e., true positives + true negatives/all observations).

Additionally, we examined the extent to which model-detected neighborhood microscale features corresponded with GIS-measured macro-level walkability and self-reported neighborhood walkability attributes (i.e., convergent validity) by conducting Spearman rank correlations between (1) model-detected microscale neighborhood features (*z*-scored individual features and total micro-scale) and GIS-measured neighborhood walkability (*z*-scored individual components and overall walkability), and (2) model-detected microscale neighborhood features (*z*-scored individual and total microscale) and participants’ NEWS items (e.g., sidewalks, curb cuts) and subscales (e.g., walking and cycling facilities).

## 3. Results

### 3.1. Image Classifier Performance

Eight image classifiers were trained to identify their respective street features (i.e., sidewalk, sidewalk buffer, curb cut, zebra crosswalk, line crosswalk, walk signal, bike symbol, and streetlight). Table 1 provides the number of images used for training and validation for each of the eight classifiers.

The performance metrics for each of the image classifiers using the Phoenix, AZ validation dataset are displayed in Table 2. Generally, accuracy was high and ranged from 99.59% for zebra crosswalks to 90.03% for streetlights. The precision values (when the model indicated the presence of a feature, how likely was the model to be correct compared to human raters) ranged from 100% for zebra crosswalks to 86.73% for sidewalk buffers. Negative predive values (i.e., when the model indicated the absence of a feature, how likely was the model to be correct compared to human raters) ranged from 99.66% for bike symbols to 89.93% for sidewalks. See Appendix A for a table of validation performance using pooled data from all five cities.

### 3.2. Model Inference Results

The prevalence of model-detected features across the 512 participant neighborhoods in Phoenix, AZ was highest for sidewalks (89.8%), followed by streetlights (31.5%), curb cuts (26.2%), sidewalk buffers (15.9%), line crosswalks (4.9%), walk signals (3.7%), bike symbols (0.5%), and zebra crosswalks (0.3%).

#### 3.2.1. Associations between Model-Detected Microscale Feature and GIS-Measured Macro-Level Walkability

Spearman correlations between model-detected microscale features and GIS-measured walkability attributes are presented in Table 3. The macroscale walkability index correlated with nine microscale features, while the four individual macroscale components correlated with seven microscale features. A general pattern of significant weak-to-moderate positive associations existed between GIS-measured macroscale walkability and microscale features (*r* = 0.11 to 0.52, *p* < 0.05). There were two exceptions to this pattern: (1) model-detected curb cuts had weak but significant negative relationships with intersection density, transit density, and overall macro-level walkability, and (2) GIS-measured intersection density had weak but significant negative associations with model-detected sidewalk buffers, curb cuts, line crosswalks, walk signals, and the total microscale feature score. Overall, the magnitude of associations with model-detected microscale features was greatest for transit density, land-use mix diversity, and overall GIS-measured walkability. Model-detected microscale features generally showing the greatest magnitude of associations with GIS-measured walkability were crosswalks, walk signals, bike symbols, streetlights, and total microscale feature scores (*r* = 0.19–0.52, *p* < 0.05).

#### 3.2.2. Associations between Model-Detected Microscale Feature and Perceived Neighborhood Walkability

Spearman correlations between model-detected microscale features and NEWS subscales walkability attributes are presented in Table 3. Subscales for perceived residential density, land-use mix diversity, presence of walking and cycling facilities, and perceived aesthetics were positively associated with one or more model-detected microscale features (*r* = 0.11–0.31). Significant negative associations were found between model-detected curb cuts and perceived residential density (*r* = −0.19, *p* = 0.000) and between model-detected sidewalks and perceived aesthetics (*r* = −0.24, *p* = 0.000). Perceived street connectivity, pedestrian safety, and crime safety were not related to any model-detected microscale feature or to the total microscale feature score. Among model-detected microscale features with corresponding individual NEWS items, there were significant positive associations between model-detected and perceived sidewalks (*r* = 0.41, *p* = 0.000), model-detected sidewalk buffers and perceived grass/dirt sidewalk buffers (*r* = 0.26, *p* = 0.000), and model-detected and perceived crosswalks and walk signals (*r* = 0.15, *p* < 0.01).

## 4. Discussion

This paper demonstrates that the use of computer vision to detect intersection and street segment features that are conceptually related to pedestrian physical activity (i.e., zebra and line crosswalks, curb ramps, walk signals, sidewalks, sidewalk buffers, bike symbols, and streetlights) is feasible with high correspondence to human raters. Individual microscale features and a summary index of microscale features correlated with both GIS-measured macroscale walkability and with human participants’ reports of their neighborhood environment. These expected correlations offer a degree of validly to the computer vision models of microscale features. The development of machine learning models for detecting microscale features opens the possibility of conducting research across broad regions and new research questions.

Computer model-detected microscale features correlated with both the individual components of macroscale walkability and the macroscale walkability index around participant neighborhoods in Phoenix. These correlations were expected, as higher levels of macroscale walkability are often complemented by improvements to more affordable microscale improvements (e.g., curb cuts, sidewalks) that further enhance the streetscape. Previous studies have shown that individual microscale features and macroscale walkability indices are weakly to moderately correlated, with both contributing unique measures of the built environment for walking [4]. While most component correlations between macro- and microscale features were positive, the macroscale component of intersection density was negatively correlated with all microscale features except sidewalks. Higher intersection densities are typically observed in denser urban settings with shorter, more connected street blocks. One could expect urban settings with shorter blocks to have a greater prevalence of sidewalks and related safety features such as crosswalks and curb cuts; however, the WalkIT AZ participants’ perceptions of street connectivity and pedestrian safety surprisingly did not correlate with any model-detected features.

Model-detected microscale features correlated with four perceived subscales of the built environment, specifically residential density, land-use mix, walking and cycling facilities, and aesthetics. WalkIT participants evaluated their neighborhoods using the previously validated NEWS, which has been validated against GIS-measured macroscale features and used in dozens of studies as a predictor of pedestrian walking for transportation with weak to moderate correlations (i.e., *r* < 0.40) [40,41]. The NEWS does not offer an index to summarize its seven subscales, but our index of model-detected microscale features did correlate with perceived residential density and walking and cycling facilities subscales (*r* = 0.13 and 0.21, respectively). The strongest microscale relationships occurred for the residential density subscale, which had eight significant correlations, with model-detected walk signals and combined crosswalks (zebra and line) correlating the strongest (*r* = 0.30–0.31). This suggests that higher levels of model-detected safety features for pedestrians correlated with higher levels of independent perceptions of residential density, which aligns with expectations that these features would be more prevalent in areas with more people. The present results were consistent with previous studies showing weak to modest agreement between objective and subjective assessments of neighborhood walkability overall, with lower concordance among those with less physical activity and higher BMI [12,51]. Because the current study included only insufficiently active individuals with a median BMI of 33.0, results may not generalize to other populations. However, considering previous research [41], we would expect higher correspondence between model-detected features and perceived neighborhood walkability in a more physically active sample.

*Methodological Considerations*. Two major strengths should be noted. First, in previous studies, human raters audited only parts of participant neighborhoods—usually limited to a quarter mile route or sample of blocks in a neighborhood—by in person or virtual observation. The current computer vision approach was used to audit all blocks and crossings for the entire neighborhood for all participants, limited only by the number of GSV photos available and the timeframe of the study. Second, WalkIT participants were purposefully recruited in similar numbers from neighborhoods high and low in walkability and high and low in neighborhood socioeconomic status. Therefore, our analyses involving microscale, macroscale, and participant perceptions reflect the full spectrum of walkability and income environments present in the Phoenix, AZ region.

However, our models were limited to seven of the 15 features assessed by the MAPS Mini roadmap. Future work will focus on developing models for detecting additional microscale features supportive of physical activity (e.g., benches important for older adults). Several MAPS Mini items, such as transit stops and public parks, are now commonly included in publicly available datasets, making it possible to conduct audits using GIS technology. Other items, such as building and sidewalk disrepair or graffiti are difficult to observe from the perspective of omnidirectional cameras, require subjective or qualitative judgement, or are more transient in nature. Alternative artificial intelligence methodologies have been suggested to overcome the scalability challenges associated with assessing these items. For example, Athens et al. [52] applied a natural language processing approach to detect sidewalk maintenance, building safety, and other urban blight indicators from 311 data, while Ping et al. [53] leveraged city garbage trucks equipped with video cameras combined with edge computing technology to develop a deep learning model for detecting and classifying street litter. A combination of approaches will likely be needed to provide a comprehensive characterization of microscale neighborhood streetscapes on a large scale. 

Several considerations also should be noted in the development of computer vision models. First, we explored existing annotated image datasets such as Mapillary [54] and CAMVID [55] and found that labeling did not capture the features of interest, as well as led to incorrect classifications and bucketing of categories, which precluded utilizing existing datasets and developing good computer vision models of the features of interest. Second, while a standardized number of images for each feature for training and validation datasets would have been conceptually clearer to report, we found that additional training or validation images were needed for certain features (e.g., bike symbols, zebra crosswalks) because of the low prevalence of such features in the Phoenix, AZ region. We also collected a greater number of training samples to ensure that we captured inherent variability in feature design and photos of features that exist in the real world in the training dataset. For example, the variability in the design of crosswalks varies even by small geographic regions to include different patterns and colors (e.g., crosswalk with LGBT rainbow flag colors). We also considered image artefacts (e.g., shadows that appear similar to zebra crossings, distance from GSV camera to streetlight) that resulted in small or fuzzy training samples. In addition, some photo elements confused the models (e.g., actual bike vs. painted bike symbol) and necessitated additional training samples. Although results presented in the current analyses used a Phoenix, AZ validation dataset, we expect that the generalizability of our trained classifiers was enhanced with the inclusion of images from four additional geographically diverse cities (i.e., Seattle, San Diego, Washington, D.C., and Baltimore) in the classifier training (see Appendix A Table A1). To scale up results to cities around the globe, images from other countries would have to be included in the training dataset, because street features such as sidewalks and crossings do not follow international standards.

## 5. Conclusions

The current results demonstrate that computer vision models can reliably conduct neighborhood audits of pedestrian streetscape features. Our model results correlate with both objective and self-reported macroscale neighborhood walkability. Future research will examine the relationship between model-detected microscale features and physical activity and chronic disease outcomes. The computer vision approach to auditing neighborhoods promises to accelerate the pace of microscale research and opens new lines of microscale research for urban planning and public health. Results suggest that automated virtual streetscape audits may provide a scalable alternative to human audits, enabling advancements in the field currently constrained by time and cost. Reducing reliance on trained auditors will enable scaling up audits to assess hundreds or thousands of neighborhoods or even entire cities for surveillance, hypothesis testing, identifying environmental disparities, or change detection research related to pedestrian streetscapes. For example, given sufficient resources, such models could be applied at scale to map all sidewalks in the US, evaluate whether motivational physical activity interventions perform better in neighborhoods with more vs. fewer sidewalks, determine whether the prevalence of sidewalks differs by neighborhoods that vary by race/ethnicity or income, or even evaluate change in sidewalks before and after a new development or passage of a complete street policy or transportation infrastructure tax. This work also has potentially important implications for urban municipality decision-makers. While previous work has largely focused on macroscale walkability due to ease of measurement, it is often not feasible to change these aspects of the built environment due to complexity and cost. However, microscale features can be more easily and cost-effectively modified than macroscale elements. Thus, large-scale microscale audits can enable municipalities to make well-informed decisions about streetscape enhancements that equitably promote physical activity within budgetary constraints. 

## Figures and Tables

**Table 1:** Summary of the number of images used for training and validation datasets.

Street Feature	Image Counts									
	Present				Absent				Total	
	All Training	All Validation	Phoenix Only Training	Phoenix Only Validation	All Training	All Validation	Phoenix Only Training	Phoenix Only Validation	Training	Validation
Sidewalk	8868	2851	5177	1745	3702	1254	2298	429	12570	4105
Sidewalk buffer	3530	629	1519	347	6066	1773	4461	1567	9596	2402
Curb cuts	5947	599	2406	268	6059	767	2459	599	12006	1366
Zebra crosswalk	1687	2456	412	100	5604	6121	2971	879	7291	8577
Line crosswalk	1762	1053	1693	758	4057	2462	3798	2257	5819	3515
Walk Signal	3126	509	1951	216	4722	1221	2747	1014	7848	1730
Bike Symbol	1127	152	853	132	9306	2138	6908	2078	10433	2290
Streetlight	1380	288	808	170	1213	273	761	171	2593	561

**Table 2:** Validation performance of image classifiers for Phoenix, AZ.

Street Feature	Performance				
	Precision	Recall	Negative Predictive Value	Specificity	Accuracy
Sidewalk	97.93%	97.48%	89.93%	91.61%	96.32%
Sidewalk buffer	86.73%	84.73%	96.63%	97.13%	94.88%
Curb cut	95.38%	92.54%	96.71%	98.00%	96.31%
Zebra crosswalk	100%	96.00%	99.55%	100%	99.59%
Line crosswalk	95.97%	94.20%	98.06%	98.67%	97.55%
Walk signals	96.77%	97.22%	99.41%	99.31%	98.94%
Bike symbols	93.28%	94.70%	99.66%	99.57%	99.28%
Streetlight	88.64%	91.76%	91.52%	88.30%	90.03%

**Table 3:** Model-detected microscale feature correlations with GIS-measured macro-level walkability and perceived NEWS scales.

Model- Detected Microscale Feature	GIS-Measured Macroscale Neighborhood Walkability					Perceived Neighborhood Walkability						
	Residential Density	Land-Use Mix Diversity	Intersection Density	Transit Density	Overall Walkability Index	Residential Density	Land-Use Mix Diversity	Street Connectivity	Walking and Cycling Facilities	Aesthetics	Pedestrian Safety	Crime Safety
Sidewalks	0.12 **	0.05	0.18 **	−0.06	0.02	−0.06	−0.02	−0.03	0.11 *	−0.24 ***	0.01	−0.02
Sidewalk Buffers	0.18 ***	0.30 ***	−0.14 **	0.01	0.17 ***	0.07 ^†^	−0.01	0.05	0.17 ***	0.19 **	−0.08 ^†^	0.01
Curb Cuts	0.04	0.16 *	−0.16 ***	−0.20 ***	−0.11 *	−0.19 ***	−0.06	0.06	0.17 ***	−0.03	0.08 ^†^	0.04
Zebra crosswalks	0.16 ***	−0.07	0.04	0.37 ***	0.02	0.15 **	0.04	−0.01	−0.04	−0.06	−0.04	−0.07
Line crosswalks	0.06	0.42 ***	−0.14 **	0.13 **	0.39 ***	0.28 ***	0.24 ***	0.01	0.02	0.03	−0.01	−0.02
All crosswalks	0.07 ^†^	0.39 ***	−0.12 **	0.38 **	0.38 ***	0.30 ***	0.23 ***	0.00	0.01	0.01	−0.01	−0.03
Walk Signals	0.09 *	0.37 ***	−0.10 *	0.52 ***	0.46 ***	0.31 ***	0.23 **	0.02	0.00	0.07	−0.07 ^†^	−0.07
Bike Symbols	0.17 **	0.22 ***	0.06	0.20 ***	0.28 ***	0.25 ***	0.15 **	−0.01	0.02	−0.03	−0.03	−0.05
Streetlights	0.23 ***	0.38 ***	0.00	0.12 **	0.35 ***	0.17 ***	0.07	−0.00	0.14 **	−0.03	−0.06	−0.07
Total Microscale	0.19 ***	0.38 ***	−0.12 *	0.11 *	0.30 ***	0.13 **	0.07 ^†^	0.02	0.21 ***	0.04	−0.02	−0.02

Notes: Spearman rank correlation coefficients. ^†^ *p* < 0.10, * *p* < 0.05, ** *p* < 0.01, *** *p* < 0.001. Model-detected features were assessed by *z*-scoring the average count of positive feature instances for coordinates within a 500 m street network buffer around participants’ homes. Perceived neighborhood features were assessed by the Neighborhood Environment Walkability Scale (NEWS). All crosswalks = sum of zebra and line crosswalks. Total microscale score = sum of *z*-score averages for bike symbols, all crosswalks, curb cuts, walk signals, sidewalks, sidewalk buffers, and streetlights within each participant’s 500 m neighborhood buffer.

## Data Availability

The data presented in this study are available on request from the corresponding author. The data are not publicly available due to ongoing data analyses related to aims of the grant.

## Appendix A

**Table A1:** Validation performance of image classifiers in pooled dataset including Phoenix AZ, San Diego CA, Washington D.C., Seattle WA, and Baltimore MD.

Street Feature	Performance				
	Precision	Recall	Negative Predictive Value	Specificity	Accuracy
Sidewalk	97.25%	96.81%	92.82%	93.78%	95.88%
Sidewalk buffer	87.10%	85.85%	95.01%	95.49%	92.96%
Curb cut	83.21%	65.86%	52.32%	73.81%	68.54%
Zebra crosswalk	97.33%	84.97%	93.61%	98.95%	94.62%
Line crosswalk	89.20%	75.59%	71.20%	86.83%	80.20%
Walk signals	86.00%	73.38%	68.80%	83.09%	77.40%
Bike symbols	95.00%	95.00%	98.33%	98.33%	97.50%
Streetlight	84.30%	86.44%	83.84%	81.37%	84.09%
